# A Rare Case of Postpartum Panhypopituitarism Without Imaging Evidence of Sheehan’s Syndrome

**DOI:** 10.7759/cureus.27413

**Published:** 2022-07-28

**Authors:** Yusra Ansari, Saad A Ansari, Tahir Muhammad Abdullah Khan, Syed Naqvi, Karen Lyons

**Affiliations:** 1 Internal Medicine, University of Kentucky, Bowling Green, USA; 2 Internal Medicine, University of California Riverside School of Medicine, Riverside, USA; 3 Pulmonary and Critical Care Medicine, University of Kentucky, Bowling Green, USA; 4 Obstetrics and Gynaecology, Med Center Health, Bowling Green, USA

**Keywords:** hyponatremia, hypothyroid, adrenal insufficiency (ai), sheehan’s syndrome, panhypopituitarism

## Abstract

We present a case of a 35-year-old female with type 2 diabetes mellitus who delivered a female neonate via normal vaginal delivery without any peripartum complication and minimal blood loss. The patient developed features of panhypopituitarism in the post-partum period with imaging with CT and MRI showing unremarkable pituitary gland. This is a rare presentation of post-partum panhypopituitarism with normal imaging studies.

## Introduction

Hypopituitarism is defined as deficiency of one or more pituitary hormones and the clinical presentation is based on the metabolic and endocrine effects of each individual hormone. Although the most common cause of postpartum panhypopituitarism is Sheehan’s syndrome (SS), it is exceedingly rare. It is usually preceded by hypovolemic shock because of significant postpartum bleeding which leads to pituitary apoplexy or necrosis. MRI of the brain may show a range of findings including pituitary enlargement with homogeneous T1 weighted hypointensity and T2 weighted hyperintensity with rim enhancement in the acute phase, followed by anterior pituitary atrophic changes during the chronic phase. Sometimes, irregular pituitary enhancement can be observed in the acute phase due to the presence of infarcted tissue interspersed with perfused tissue [1]. We report a diagnostically challenging case of post-partum panhypopituitarism in a patient with no preceding hemorrhagic or hypovolemic shock and with no imaging evidence of pituitary abnormality. 

## Case presentation

A 35-year-old female gravida 4, para 3 with a past medical history of type 2 diabetes mellitus presented at 33 weeks of gestation with regular uterine contractions. She was alert and awake and her vitals were within normal range with normal fetal examination as expected for her gestational age. She was found in active premature labor and was admitted to the obstetric floor for further management. She had a vaginal delivery of a female neonate without any peripartum complication. Epidural anesthesia was given during delivery, and approximately 300 mL of blood loss was reported with no hemodynamic instability. She started complaining of a mild headache within 24 h of delivery. She was discharged home after 24 h of observation and was advised to use acetaminophen for headaches. 

She presented on the 10th day post-partum with a complaint of severe occipital headache associated with nausea and vomiting and her symptoms were progressing over one week. She had decreased oral intake for the last 3 days due to vomiting and she was having mild cold intolerance, generalized weakness, and dizziness. She denied focal motor or sensory deficits, diplopia, or vision abnormalities. She was able to lactate her infant. She was alert, awake, and oriented but she looked anxious about her health and a flat affect was noticed. She did not have a fever, chills, abdominal pain, constipation, or diarrhea. She was afebrile with a blood pressure of 122/79 mmHg, heart rate of 71 beats/min, and oxygen saturation of 98% on room air. Laboratory tests of the patient are given in Table 1.

**Table 1:** Laboratory work-up. ACTH, adrenocorticotrophic hormone

Laboratory parameter	Patient’s results	Normal range
Serum sodium	109 mEq/L	137-145 mEq/L
Serum potassium	3.2 mEq/L	3.5-5.1 mmol/L
Serum creatinine	0.56 mg/dL	0.52-1.04 mg/dL
Serum bicarbonate	22 mmol/L	22-30 mmol/L
Alanine aminotransferase	184 U/L	0-34 U/L
Aspartate aminotransferase	226 U/L	14-36 U/L
Total bilirubin	0.6 mg/dL	0.2–1.3 mg/dL
Serum glucose	112 mg/dL	74–106 mg/dL
Urine sodium	117 mmol/L	30–90 mmol/L
Urine osmolality	328 mosm/kg	300–1000 mosm/Kg
Serum osmolality	234 mosm/kg	286–309 mosm/Kg
Thyroid stimulating hormone	0.299 mIU/L	0.465–4.680 mIU/L
Free T3	1.97 pg/mL	2.77–5.27 pg/mL
Free T4	0.35 ng/dL	0.78–2.19 ng/dL
Serum morning cortisol	4.1 mcg/dL	4.46–22.7 mcg/dL
Serum ACTH	19.7 pg/mL	7.2–63.3 pg/mL

Her lab work showed a normal complete blood count. The basic metabolic profile showed severe hyponatremia with mild transaminitis. She appeared euvolemic on examination, was not on any diuretics, and did not have any complaints of polydipsia or polyuria. She had normal mentation with no seizure activity. Further work up to investigate her hyponatremia was done. Urine osmolality was high with high urine sodium and low serum osmolality. She also had central hypothyroidism and adrenal insufficiency with inappropriately low serum cortisol and adrenocorticotropic hormone (ACTH). Her presentation of central hypothyroidism, adrenal insufficiency, and symptomatic hyponatremia with complaints of headaches since delivery was concerning for panhypopituitarism. CT scan brain and later MRI brain were performed that was unremarkable for empty sella, pituitary apoplexy, hemorrhage, necrosis, and enlargement. She was started on IV hydrocortisone 50 mg every 8 h, and she was given IV levothyroxine 100 mcg once. Later she was started on oral hydrocortisone 10 mg twice daily and oral levothyroxine 100 mcg once daily. She became clinically better and showed improvement in her lab work. Serum sodium became normal after replacement and liver function tests trended down. She was discharged on oral levothyroxine 100 mcg once daily and oral hydrocortisone 10 mg twice daily with a follow-up arranged with her primary care and endocrinologist. On follow-up with the endocrinologist 10 days after discharge, she was symptom-free and was able to lactate her neonate. The patient was compliant with levothyroxine and hydrocortisone, and repeat labs showed thyroid stimulating hormone (TSH) of 0.778 mIU/L (0.465-4.680 mIU/L), free T4 of 0.71 ng/dL (0.78-2.19 ng/dL), free T3 of 1.97 pg/mL (2.77-5.27 pg/mL), and serum cortisol level in the afternoon was 2.1 mcg/dL (3-10 mcg/dL). She was advised to continue her current medications and the next follow-up was scheduled in 4 weeks with a repeat TSH and morning cortisol levels. 

## Discussion

Sheehan's syndrome is pituitary necrosis after hemodynamic instability due to post-partum hemorrhage or hypovolemia and occurs in 1%-2% of women with loss of 1-2 L of blood volume and associated hypotension. Although hypotension and hemorrhage leading to anemia with a drop in hemoglobin between 3 and 8 g/dL have been the most reported causative factor associated with SS, there are a few cases of it reported in literature involving patients who did not have these risk factors. In one report, the patient developed SS with only 500 mL of blood loss in the peripartum period [2]. Our patient had only 300 mL of blood loss.

Pregnancy is associated with normal physiological changes in the endocrine system required for fetal development and preparation for labor. The pituitary gland is one of the most affected organs to develop anatomical and physiological changes. Estrogen-mediated lactotroph hyperplasia occurs with a 40% increase in the lactotroph tissue and leads to hyperprolactinemia with prolactin levels as high as 200 ng/mL. The role of progesterone on prolactin secretion has also been suggested. Although baseline serum prolactin levels are elevated, responses to stimuli like thyrotropin-releasing hormone and sleep are maintained. Associated with these changes, the pituitary gland also becomes more vascularized and, in the presence of estrogen, more blood supply to the pituitary is derived from the systemic circulation, which has a low dopamine concentration, and a lesser share of blood supply is from the hypothalamic-pituitary portal circulation that is high in dopamine content [3]. At the time of delivery, any sudden drop in blood pressure or low hemoglobin due to blood loss can adversely affect hypertrophied and hypervascularized anterior pituitary gland tissue leading to ischemic necrosis. 

Sheehan’s syndrome can present with hypopituitary symptoms either within a few weeks of the insult or can have subtle manifestations leading to a delayed diagnosis years later from the insult. It can have a range of presentations with the most common electrolyte abnormality on lab work being hyponatremia which is usually secondary to adrenal insufficiency or central hypothyroidism. Both adrenal insufficiency and central hypothyroidism can cause decreased free water clearance leading to hyponatremia. Adrenal insufficiency also causes an increase in antidiuretic hormone contributing to hyponatremia [4-5]. The onset of a severe headache on the day of delivery has been reported as a sign of SS, however, it is a nonspecific symptom as other etiologies like tension headache, post-dural puncture headache, migraines, cerebral venous thrombosis, and intracranial hemorrhage can present with acute headache, and it needs to be investigated with imaging of the brain [6]. Our patient did have an acute onset of headache on the day of delivery, and it persisted with variable intensity till diagnosis was made, and she had other associated symptoms of generalized weakness, nausea, and vomiting which were attributed to severe hyponatremia due to adrenal and thyroid deficiency. Other causes of panhypopituitarism that were considered in our patient were lymphocytic hypophysitis, hemochromatosis, and neurosarcoidosis. Hemochromatosis is extremely rare in women of childbearing age due to iron loss during menstruation, and normal MRI findings with no previous history of autoimmune disease made lymphocytic hypophysitis and neurosarcoidosis unlikely in our patient. The most common MRI findings in the acute phase of SS are enlarged pituitary gland with the abnormal (hypointense) signal on T1 weighted images and hyperintense signal on T2 weighted images with rim enhancement [1]. In a case series of SS, most common brain MRI findings were empty sella of normal size which was observed in 11 of 13 patients [7]. SS presenting with normal MRI findings is rare.

The clinical course of SS is variable and ranges from mild disease to permanent hypopituitarism, and these patients need hormone replacement therapy. Close monitoring of the clinical condition and follow-up lab work are warranted to determine hormone deficiency requiring replacement. Also, women with SS should be counseled about future pregnancy and obstetric implications related to SS with hypopituitarism as it is associated with an increased risk of obstetric complications. 

## Conclusions

Sheehan’s syndrome is a rare but potentially life-threatening complication, and a low threshold should be kept in considering a diagnosis of SS in those postpartum patients who develop moderate to severe headache within 24 h of delivery irrespective of the presence of any preceding event of large blood loss or hypotension. 
